# Assessment of Ocular Torsion in Exotropic Patients Following Horizontal Strabismus Surgery: A Comparative Analysis Using Conventional Color Fundus Photography and Spectralis Optical Coherence Tomography

**DOI:** 10.1155/joph/1477145

**Published:** 2025-09-07

**Authors:** Kaveh Abri Aghdam, Zahra-Alsadat Abtahi, Amin Zand, Mostafa Soltan Sanjari, Ali Sadeghi, Vahid Zare Hosseinabadi, Seyed Ali Sonbolestan

**Affiliations:** ^1^Eye Research Center, Ophthalmology Department, Moheb Kowsar Hospital, The Five Senses Health Institute, School of Medicine, Iran University of Medical Sciences, Tehran, Iran; ^2^Department of Ophthalmology, Shafa Hospital, Kerman University of Medical Sciences, Kerman, Iran; ^3^Department of Oculo-Facial Plastic and Reconstructive Surgery, Farabi Hospital, Eye Research Center, Tehran University of Medical Sciences, Tehran, Iran

**Keywords:** disc-fovea angle (DFA), exotropia, fovea-to-disc (FoDi) alignment software, fundus photography, horizontal strabismus surgery, ocular torsion, Spectralis optical coherence tomography (OCT)

## Abstract

**Purpose:** To objectively evaluate ocular cycloposition using conventional color fundus photography (CFP) and Spectralis optical coherence tomography (OCT) in exotropic patients undergoing horizontal strabismus surgery and to assess the agreement between these methods. Additionally, we aimed to determine whether surgery alters ocular torsion in pure exotropia.

**Methods:** In this prospective, single-center study, 42 eyes from 21 patients with exotropia requiring bilateral lateral rectus muscle recession were evaluated. The disc-center fovea angle (DFA) was measured pre- and postoperatively using CFP (Topcon fundus camera) and Spectralis OCT with fovea-to-disc (FoDi) alignment software.

**Results:** The angle of exodeviation improved significantly postoperatively, from 35.10 ± 7.89 to 3.76 ± 3.73 prism diopters (*p* < 0.001). Both CFP- and OCT-derived DFA values remained stable in both eyes at 6 months (*p* > 0.05 for all comparisons). No significant differences were observed between CFP and OCT measurements at baseline or postoperatively (*p* > 0.05). Preoperative intraclass correlation coefficient (ICC) between methods was strong (right eye: 0.765; left eye: 0.750), decreasing postoperatively to moderate levels (right eye: 0.618; left eye: 0.661).

**Conclusion:** Horizontal strabismus surgery does not significantly alter ocular torsion in pure exotropia. Spectralis OCT reliably quantifies cyclotorsion but may yield marginally lower DFA values compared to CFP, despite no statistical difference.

## 1. Introduction

The cycloversion mechanism plays a critical role in maintaining clear vision and binocular single vision, primarily regulated by the vestibular system to stabilize retinal images during head movements [1]. While human binocular vision can fuse torsional disparities up to 10° through cyclofusion, deviations exceeding 6° significantly impair horizontal fusional vergence and stereopsis [2]. Studies suggest that the degree of ocular torsion, influenced by fusional control, may predict responses to strabismus surgery [3]. Abnormal ocular torsion is typically associated with dysfunction of the extraocular muscles, particularly those involved in oblique muscle actions [4]. However, it has also been documented in horizontal strabismus, including intermittent exotropia [5]. Accurate preoperative assessment of torsion in these cases may refine surgical planning and improve postoperative outcomes.

While traditional tests like the double Maddox rod or Lancaster red-green test can assess ocular torsion, their subjective nature often yields unreliable results in uncooperative patients or chronic strabismus cases. Therefore, objective measurements are essential for accurate diagnosis and surgical planning in cyclovertical strabismus. Established objective methods include measuring torsional angles via indirect ophthalmoscopy or fundus photography [6]. Among these, the disc-center fovea angle (DFA), widely recognized as a gold-standard metric for quantifying ocular torsion [7], is traditionally derived from fundus photographs. Recent advances in optical coherence tomography (OCT) now enable automated DFA calculation, offering enhanced precision and efficiency [8].

The Spectralis platform of spectral domain OCT utilizes an exclusive feature, fovea-to-disc (FoDi) alignment, to evaluate the position of the fovea in relation to the center of the optic disc. This technology enhances the accuracy and reproducibility of retinal nerve fiber layer (RNFL) thickness measurements, allowing automatic determination of the DFA using FoDi software [8, 9]. Color fundus photography (CFP) was performed using a Topcon fundus camera with a 45° field of view. While a limited number of studies have objectively evaluated ocular torsion in eyes with horizontal strabismus [3, 10], this study had two primary objectives: first, to compare the accuracy of DFA measurements obtained via CFP and Spectralis OCT in patients with exotropia; and second, to evaluate whether horizontal strabismus surgery (bilateral lateral rectus muscle recession) influences ocular torsion in this patient population. Establishing the reliability of OCT as a tool for assessing cyclotorsion could streamline clinical workflows by reducing reliance on pupillary dilation and external image analysis, thereby enhancing surgical planning and postoperative assessment.

## 2. Materials and Methods

This prospective, single-center observational study was conducted at a tertiary pediatric ophthalmology and strabismus clinic of Rasool-e-Akram Hospital, Iran University of Medical Sciences, Tehran, Iran, between January 2022 and August 2023. The study adhered to the ethical principles of the Declaration of Helsinki and received approval from the Institutional Ethics Committee of the Iran University of Medical Sciences. Written informed consent was obtained from all participants or their legal guardians prior to enrollment.

### 2.1. Study Population

The study enrolled patients diagnosed with exotropia undergoing primary bilateral lateral rectus muscle recession. Eligible participants exhibited a minimum exodeviation of 15 prism diopters (PDs) at both distance (6 m) and near (33 cm) fixation, confirmed via prism and alternate cover testing (Prism Bar Set, Luneau Technology, France).

Exclusion criteria comprised ocular motility abnormalities such as vertical deviation in primary gaze, A/V patterns, nonconcomitant strabismus (restrictive or paretic), or dissociated deviations; ocular comorbidities including optic disc anomalies (tilt, coloboma, and hypoplasia), macular pathology (edema, dragging, and scarring), or spherical equivalent refractive errors exceeding ±6.00 diopters; prior ocular surgery (e.g., strabismus repair, retinal detachment, and orbital fracture); and technical limitations such as inadequate fundus image quality precluding foveal reflex assessment.

### 2.2. Study Protocol

At each visit, patients underwent a comprehensive ophthalmic examination, including cover-uncover and alternate cover tests to evaluate ocular alignment at both near (33 cm) and distance (6 m) fixation. All strabismus assessments, including the cover-uncover test, alternate cover test, and prism measurements, were performed by a single experienced pediatric ophthalmologist to minimize interobserver variability. To ensure the accuracy of our data, all strabismus assessments were initially performed by a single experienced pediatric ophthalmologist (KAA). The protocol was standardized so that measurements were taken three times, with the mean value being recorded. Subsequently, all angles of deviation data were independently double-checked by a second ophthalmologist to confirm accuracy. A discrepancy was defined as a difference exceeding 2 PDs between the two assessments. In cases where such a discrepancy occurred, the two ophthalmologists performed a joint reevaluation of the patient to reach a definitive consensus on the measurement, which was then recorded as the final value. This joint reevaluation served as the retesting mechanism to ensure agreement. After the surgical procedure on the horizontal rectus muscles, follow-up visits were scheduled at 1 week, 1 month, 3 months, and 6 months postoperatively. Cyclotorsion was evaluated both preoperatively and at the 6-month mark using CFP (Topcon fundus camera, Topcon, Japan) and a peripapillary RNFL program via SD-OCT (Spectralis OCT, Heidelberg Engineering, Germany).

### 2.3. Surgical Procedure

All bilateral lateral rectus muscle recession surgeries were performed by the same ophthalmologist who conducted the preoperative assessments. Surgical dosages were determined preoperatively using a standardized surgical table based on the angle of exodeviation, patient age, and historical motor response data. Adjustable sutures were utilized in every case to facilitate precise positioning of the muscles. Under topical anesthesia, these sutures were adjusted within the first 24 h postoperatively for optimal alignment.

### 2.4. Objective Torsion Assessment

Objective ocular torsion in both eyes of each patient was assessed using CFP and Spectralis OCT. Each eye was evaluated individually in the primary position. For both modalities, patients were instructed to fixate on an internal target while maintaining stable head alignment with a chin and forehead rest to prevent tilting. During testing, both eyes remained open to replicate natural binocular conditions [10, 11].

**Figure 1:**
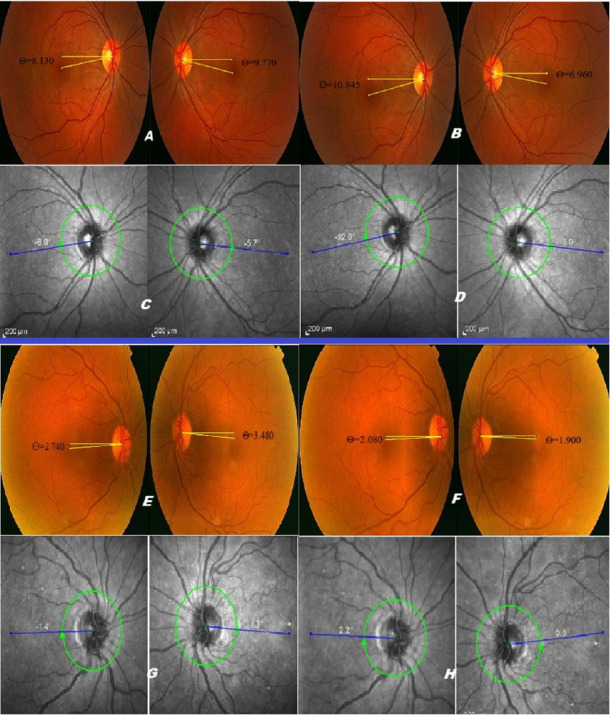
Preoperative and postoperative ocular torsion measurements in two representative patients with exotropia, using conventional color fundus photography (CFP) and Spectralis optical coherence tomography (OCT). The top two rows display images for the first patient. (A) (preoperative) and (B) (postoperative) show CFP images; (C) (preoperative) and (D) (postoperative) show OCT images. The bottom two rows display images for the second patient, following the same layout: (E) (preoperative) and (F) (postoperative) for CFP and (G) (preoperative) and (H) (postoperative) for OCT. Within all panels, the image of the right eye (OD) is on the left, and the left eye (OS) is on the right. Ocular torsion is quantified as the disc-center-fovea angle (DFA), represented by the angle (ϴ) between a horizontal reference line and a line connecting the centers of the optic disc and the fovea.

Following mydriatic CFP, objective quantitative ocular torsion was evaluated using ImageJ software (National Institute of Health, Bethesda, Maryland, USA). The central point of the optic disc was defined as the intersection of its horizontal and vertical diameters (Figure 1) [7, 10]. Subsequently, the DFA was calculated as the angle (in degrees) between the line connecting the optic disc center to the foveal reflex and the horizontal line passing through the optic disc center (Figure 1). Extorsion was assigned a positive value, and intorsion a negative value.

During the same visit, patients underwent Spectralis OCT imaging. DFA values for each eye were recorded using the FoDi alignment software, following established protocols [8, 9]. Importantly, the Spectralis OCT system employs automated torsional eye position adjustments during follow-up imaging, aligning subsequent scans to baseline references [12]. To mitigate potential bias from this feature, patient identification codes were systematically anonymized during follow-up analyses, ensuring direct and unbiased comparisons of pre- and postoperative data.

### 2.5. Statistical Analysis

Statistical analyses were conducted using SPSS Version 23.0 (SPSS Inc., Armonk, NY). Continuous variables were expressed as mean ± standard deviation (SD), and categorical variables were expressed as percentages. To account for the nonindependence of measurements from the two eyes of the same patient, linear mixed-effects models were used to evaluate changes in objective ocular torsion angles pre- and postoperatively, as well as differences between measurements obtained from CFP and OCT. The validity of OCT-derived torsion values was assessed by correlating them with CFP measurements using the intraclass correlation coefficient (ICC), calculated with a two-way random-effects model (absolute agreement for single measurements) and interpreted via Altman's estimation method [13]. Systematic differences (bias) and 95% limits of agreement (LoA) between the two methods were analyzed using Bland–Altman plots [14]. A *p* value less than 0.05 was considered statistically significant.

## 3. Results

The study included 42 eyes of 21 patients with exotropia (12 males [57.1%], 9 females [42.9%]), with a mean age of 21.86 ± 12.07 years (range: 9–77). Preoperatively, the mean exodeviation angle was 35.10 ± 7.89 PD, which significantly decreased to 3.76 ± 3.73 PD 6 months postoperatively (*p* < 0.001). DFA values measured preoperatively by CFP were 5.33 ± 3.61° (right eye) and 6.36 ± 3.79° (left eye), while Spectralis OCT yielded values of 4.59 ± 3.14° (right eye) and 5.01 ± 3.70° (left eye). Postoperatively, ocular torsion remained stable, with no significant differences from baseline in either CFP (right eye: 4.70 ± 2.99°, left eye: 7.00 ± 3.62°) or OCT measurements (right eye: 4.11 ± 3.18°, left eye: 6.00 ± 3.98°; all *p* > 0.05; Table 1).

At baseline, ocular torsion measurements derived from CFP and Spectralis OCT showed no significant differences in either the right (*p*=0.260) or left eye (*p*=0.071). Similarly, 6 months postoperatively, no significant discrepancies were observed between the two methods for the right (*p*=0.415) or left eye (*p*=0.242; Table 1).

Preoperatively, ICC values between CFP and OCT demonstrated strong agreement (right eye: 0.765; left eye: 0.750), aligning with Altman's criteria for good correlation [13].

Postoperatively, however, correlations decreased to moderate levels (right eye: 0.618; left eye: 0.661).

**Figure 2:**
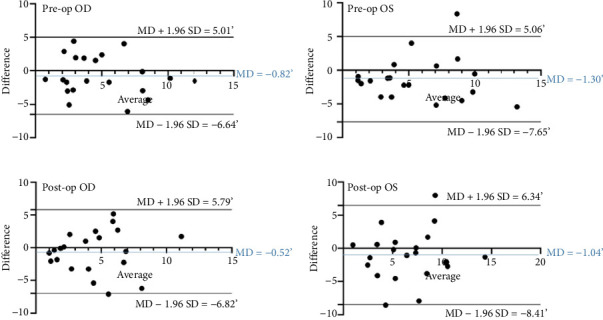
Bland–Altman plots assessing agreement between optical coherence tomography (OCT) and conventional color fundus photography (CFP) measurements of ocular torsion (in degrees) in the right eye (OD) and left eye (OS) during preoperative and postoperative evaluations. The vertical (*Y*) axis represents the difference between OCT and CFP values (OCT minus CFP), while the horizontal (*X*) axis displays the mean of paired measurements. The solid blue line indicates the mean difference (MD), and dashed black lines denote the 95% limits of agreement (MD ± 1.96 SD). SD: standard deviation.

Bland–Altman analysis revealed no systematic bias between CFP and OCT for either eye, pre- or postoperatively. The 95% LoA for preoperative measurements were ±5.83° (right eye) and ±6.31° (left eye), widening postoperatively to ±6.36° (right) and ±7.38° (left). These results align with the ICC findings, reflecting stronger preoperative agreement compared to postoperative assessments (Figure 2).

## 4. Discussion

Ocular torsion can be assessed through subjective or objective methodologies. Objective techniques quantify the rotational angle of the globe by measuring the anatomical relationship between the fovea and optic disc. The gold-standard method for evaluating objective ocular torsion involves calculating the DFA via fundus photography, which provides precise and reproducible measurements of torsional alignment [7].

In this study, we aimed to compare DFA measurements obtained via CFP and Spectralis OCT in patients with exotropia, both before and after bilateral lateral rectus muscle recession. Our results demonstrated no significant differences in cyclotorsional angles between CFP and OCT measurements (*p* > 0.05), and no clinically meaningful changes in objective ocular torsion were observed postoperatively.

The Spectralis OCT system (Heidelberg Engineering) incorporates FoDi alignment software, which semiautomatically determines the disc-fovea axis orientation during RNFL analysis [9]. Previous studies have validated this software's reliability in orthotropic eyes, showing strong agreement with CFP-derived measurements (ICC = 0.71) [8, 15]. Notably, our preoperative ICC values (right eye: 0.765; left eye: 0.750) align closely with these findings, further supporting OCT as a robust tool for torsion assessment. Similarly, Kang et al. [10] confirmed OCT's validity for quantifying cyclotorsion in intermittent exotropia, consistent with our comparative analysis against CFP.

Key advantages of OCT over CFP include its ability to eliminate the need for pupillary dilation, bypass external image processing for cycloposition measurement, and precisely localize the fovea [8].

The normal DFA ranges from 0° to 12°–13° when measured via CFP [16]. Prior studies using OCT in orthotropic eyes have corroborated these normative DFA values, demonstrating consistency between OCT and CFP methodologies [8, 9, 17]. While pathological ocular torsion is well-documented in torsional or vertical strabismus, few studies report its presence in purely horizontal strabismus [3, 18]. For instance, Khanna et al. [18] evaluated patients with infantile esotropia and observed a high prevalence of pathological objective torsion. In our study, however, the preoperative ocular torsion levels were within the range typically seen in orthotropic eyes. Similarly, Kang et al. [10] reported comparable DFA values in eyes with intermittent exotropia, with the right and left eyes measuring 5.70 ± 3.35° and 6.37 ± 3.36°, respectively, via conventional CFP, and 5.73 ± 3.61° and 6.16 ± 3.50° via OCT.

Extorsion (excyclotorsion) is the predominant torsional subtype in horizontal strabismus [3, 18, 19]. All eyes in our cohort exhibited extorsion, consistent with Lee et al. [3] who reported bilateral excyclotorsion in 96.6% of intermittent exotropia cases. However, our study found no significant change in cyclotorsion following bilateral lateral rectus muscle recession, diverging from Lee et al.'s [3] study, who observed a postoperative reduction in summed bilateral torsion (15.8 ± 4.6° vs. 13.7 ± 5.1°, *p* < 0.001). This discrepancy likely stems from methodological differences: Lee et al. [3] analyzed the cumulative torsion of both eyes, whereas our study evaluated individual ocular torsion, yielding comparatively lower baseline values.

The Spectralis OCT system (Heidelberg Engineering) employs TruTrack Active Eye Tracking, a feature that creates a baseline retinal map during initial imaging. Subsequent scans utilize AutoRescan to precisely align follow-up images with this map, minimizing operator-dependent positioning errors and achieving micrometer-level reproducibility [20]. To mitigate potential bias from automated adjustments, follow-up data were analyzed independently of baseline references.

While mydriatic fundus photography was used in this study, dilated pupils may induce subtle ocular rotation due to compromised fixation. Nonmydriatic imaging, conversely, preserves physiological conditions and improves fixation stability [10, 21]. Despite this, Kang et al. [10] reported comparable DFA values in intermittent exotropia using nonmydriatic CFP, aligning with our results. Yamadera et al. [22] further demonstrated strong preoperative and postoperative agreement between CFP and OCT in diverse excyclotorsion etiologies. In contrast, our cohort—exclusively exotropic with excyclotorsion—showed strong preoperative OCT-CFP correlation (ICC = 0.765–0.750) but moderate postoperative agreement (ICC = 0.618–0.661), suggesting surgery-induced variability.

Study limitations include a modest sample size (*n* = 21), broad age range (9–77 years), and heterogeneous preoperative exodeviation angles (20–50 PD), which may influence measurement precision. However, prior studies [3, 10, 23, 24] indicate that age does not significantly affect DFA, supporting the robustness of our torsion assessments. The modest sample size inherently limits the statistical power of our study, increasing the risk of a Type II error. Consequently, our finding of no significant postoperative change in ocular torsion should be interpreted with caution, as a subtle but real effect may exist that our study was underpowered to detect.

Furthermore, the limited cohort size impacts the reliability of longitudinal and subgroup comparisons. Trends observed over the follow-up period or within potential subgroups (e.g., based on age or the initial magnitude of deviation) may be influenced by individual outliers and lack statistical robustness. This also constrains the generalizability of our findings, as our single-center cohort may not fully represent the broader population of patients with exotropia.

To address these limitations, we are actively continuing patient recruitment to expand this dataset. Our future plans include conducting a follow-up study with a larger, multicenter cohort. This approach will not only enhance the statistical power and generalizability of our conclusions but will also enable more meaningful subgroup analyses to better investigate the potential influence of variables such as patient age and the size of the strabismus deviation on postoperative torsional outcomes.

## 5. Conclusion

In patients with pure exotropia, horizontal strabismus surgery does not significantly alter ocular torsion. Compared to CFP, the Spectralis OCT's FoDi software may yield marginally lower DFA values. However, FoDi provides a clinically efficient alternative, eliminating the need for pupillary dilation and manual image analysis while maintaining diagnostic accuracy. Its automated workflow enhances practicality for routine torsional assessments in exotropia management.

## Figures and Tables

**Table 1:** Preoperative and postoperative measurements of ocular torsion using color fundus photography (CFP) and Spectralis optical coherence tomography (OCT).

Time of evaluation	Angle of exotropia (prism diopters)	Eye	CFP (degrees)	OCT (degrees)	*p* (CFP vs. OCT)
Baseline	35.09 ± 7.88	Right	5.33 ± 3.61	4.59 ± 3.14	0.260
		Left	6.36 ± 3.79	5.01 ± 3.70	0.071

Month 6	3.76 ± 3.72	Right	4.70 ± 2.99	4.11 ± 3.18	0.415
		Left	7.00 ± 3.62	6.00 ± 3.98	0.242

*p* (baseline vs. month 6)	< 0.001	Right	0.301	0.486	
		Left	0.353	0.184	

Abbreviations: CFP = color fundus photography; OCT = optical coherence tomography.

## Data Availability

The authors confirm that the data supporting the findings of this study are available within the article and its supporting information. The participants' detailed data are available on request from the corresponding author, Seyed Ali Sonbolestan. These data are not publicly available due to the possibility of compromising the privacy of research participants.

## Supporting Information

Supporting Tables 1 and 2 summarize the clinical characteristics and surgical outcomes in exotropia patients and preoperative and 6-month postoperative ocular torsion measurements in both eyes of each patient, evaluated using conventional color fundus photography (CFP) and Spectralis optical coherence tomography (OCT), respectively.
