# Supplementary material for: Assessment of Ocular Torsion in Exotropic Patients Following Horizontal Strabismus Surgery: A Comparative Analysis Using Conventional Color Fundus Photography and Spectralis Optical Coherence Tomography
Source: J Ophthalmol. 2025 Sep 7;2025:1477145. doi: 10.1155/joph/1477145 (PMC12433727; doi:10.1155/joph/1477145)
Supplement: Supporting Information — Additional supporting information can be found online in the Supporting Information section. [file 1477145.f1.docx]

**Supplementary Table 1.** Clinical characteristics and surgical outcomes in exotropia patients.

| Patient Number | Age  (years) | Gender | Exotropia Type | Prior Treatments | Pre-op Exodeviation (PD) | SE Refractive Error (Right, D) | SE Refractive Error (Left, D) | Right LR Recession (mm) | Left LR Recession (mm) | Post-op Exodeviation (PD) |
| --- | --- | --- | --- | --- | --- | --- | --- | --- | --- | --- |
| 1 | 4 | Female | X (T) | Glasses | 34 | -1.50 | -1.75 | 7.5 | 6.5 | 1w: 12; 1m: 8; 3m: 6; 6m: 4 |
| 2 | 6 | Male | X (T) | None | 20 | +0.75 | +1.00 | 6.5 | 6.0 | 1w: 10; 1m: 6; 3m: 4; 6m: 0 |
| 3 | 6 | Female | Constant | Glasses + Patching | 45 | -3.00 | -3.25 | 8.5 | 8.5 | 1w: 16; 1m: 10; 3m: 8; 6m: 6 |
| 4 | 6 | Female | X (T) | Glasses | 28 | -2.25 | -2.50 | 7.0 | 5.5 | 1w: 12; 1m: 8; 3m: 4; 6m: 2 |
| 5 | 8 | Female | X (T) | Glasses + Orthoptic Therapy | 42 | -4.50 | -4.75 | 7.5 | 8.5 | 1w: 14; 1m: 10; 3m: 6; 6m: 6 |
| 6 | 10 | Male | X (T) | Patching | 22 | +0.50 | +0.25 | 6.0 | 5.5 | 1w: 10; 1m: 6; 3m: 2; 6m: 0 |
| 7 | 10 | Female | Constant | Glasses | 45 | -5.00 | -5.25 | 9.0 | 9.0 | 1w: 14; 1m: 8; 3m: 6; 6m: 5 |
| 8 | 11 | Male | X (T) | Glasses | 36 | -1.00 | -1.25 | 6.5 | 7.5 | 1w: 10; 1m: 6; 3m: 4; 6m: 2 |
| 9 | 13 | Female | X (T) | Orthoptic Therapy | 38 | +2.00 | +1.75 | 7.5 | 7.5 | 1w: 12; 1m: 8; 3m: 4; 6m: 2 |
| 10 | 17 | Male | X (T) | Glasses | 40 | -3.75 | -4.00 | 7.5 | 8.5 | 1w: 14; 1m: 10; 3m: 6; 6m: 6 |
| 11 | 17 | Male | X (T) | Orthoptic Therapy | 26 | +0.25 | +0.50 | 5.5 | 6.5 | 1w: 8; 1m: 4; 3m: 2; 6m: 0 |
| 12 | 18 | Male | Constant | Glasses | 42 | -5.50 | -5.75 | 9.0 | 8.0 | 1w: 18; 1m: 12; 3m: 10; 6m: 8 |
| 13 | 21 | Male | X (T) | Glasses | 30 | -2.75 | -3.00 | 6.0 | 7.0 | 1w: 10; 1m: 6; 3m: 4; 6m: 2 |
| 14 | 23 | Male | X (T) | Glasses + Orthoptic Therapy | 32 | -1.25 | -1.50 | 7.5 | 6.5 | 1w: 12; 1m: 8; 3m: 6; 6m: 2 |
| 15 | 24 | Male | Constant | Glasses | 46 | -4.00 | -4.25 | 8.5 | 8.5 | 1w: 16; 1m: 10; 3m: 8; 6m: 8 |
| 16 | 26 | Female | X (T) | None | 24 | +1.50 | +1.25 | 6.0 | 7.0 | 1w: 10; 1m: 6; 3m: 2; 6m: 0 |
| 17 | 27 | Male | X (T) | Glasses | 34 | -0.75 | -1.00 | 7.0 | 6.0 | 1w: 12; 1m: 8; 3m: 4; 6m: 2 |
| 18 | 42 | Male | X (T) | Glasses | 36 | -2.50 | -2.75 | 7.0 | 8.0 | 1w: 14; 1m: 8; 3m: 6; 6m: 4 |
| 19 | 43 | Female | Constant | Glasses | 50 | -5.25 | -5.50 | 9.0 | 9.0 | 1w: 18; 1m: 12; 3m: 10; 6m: 10 |
| 20 | 50 | Female | Constant | None | 28 | +0.00 | +0.25 | 6.5 | 5.5 | 1w: 10; 1m: 6; 3m: 2; 6m: 0 |
| 21 | 77 | Male | Constant | Glasses | 38 | -3.50 | -3.75 | 7.0 | 8.0 | 1w: 14; 1m: 10; 3m: 6; 6m: 6 |

D: diopter; m: month(s); PD: prism diopter; SE: spherical equivalent; w: week; X(T): intermittent exotropia

**Supplementary Table 2.** Preoperative and 6-month postoperative ocular torsion measurements in both eyes of each patient, evaluated using conventional color fundus photography (CFP) and Spectralis optical coherence tomography (OCT).

| Patient Number | Preoperative OD-CFP | Preoperative OS-CFP | Preoperative OD-OCT | Preoperative OS-OCT | Postoperative  OD-CFP  (6-mo) | Postoperative  OS-CFP  (6-mo) | Postoperative  OD-OCT  (6-mo) | Postoperative  OS-OCT  (6-mo) |
| --- | --- | --- | --- | --- | --- | --- | --- | --- |
| 1 | 1.38 | 2.87 | 0.10 | 1.30 | 4.34 | 11.90 | 1.10 | 9.20 |
| 2 | 9.98 | 15.95 | 3.90 | 10.50 | 7.10 | 14.99 | 1.70 | 13.70 |
| 3 | 9.57 | 2.39 | 6.60 | 0.40 | 9.10 | 8.55 | 2.00 | 0.00 |
| 4 | 4.28 | 9.65 | 1.40 | 4.50 | 7.85 | 11.62 | 5.60 | 3.70 |
| 5 | 4.65 | 4.87 | 8.70 | 0.90 | 5.66 | 5.50 | 2.40 | 1.40 |
| 6 | 0.67 | 11.45 | 5.10 | 8.20 | 3.88 | 10.45 | 7.90 | 6.60 |
| 7 | 4.61 | 6.78 | 3.10 | 7.40 | 1.57 | 5.17 | 3.60 | 5.00 |
| 8 | 3.93 | 11.31 | 0.90 | 6.90 | 1.59 | 7.18 | 1.20 | 11.30 |
| 9 | 2.71 | 1.91 | 4.60 | 0.40 | 3.30 | 4.80 | 4.30 | 5.70 |
| 10 | 5.07 | 5.73 | 0.00 | 3.50 | 2.51 | 7.52 | 0.70 | 3.00 |
| 11 | 3.19 | 7.85 | 1.50 | 9.50 | 3.30 | 7.70 | 5.80 | 9.40 |
| 12 | 10.71 | 6.12 | 9.60 | 3.90 | 4.93 | 3.72 | 7.60 | 1.20 |
| 13 | 8.13 | 9.77 | 8.00 | 5.70 | 10.27 | 6.94 | 12.00 | 5.90 |
| 14 | 3.75 | 4.00 | 5.30 | 2.80 | 4.07 | 3.39 | 5.60 | 2.00 |
| 15 | 3.81 | 5.69 | 6.20 | 1.70 | 3.35 | 7.33 | 8.50 | 7.40 |
| 16 | 12.81 | 3.19 | 11.30 | 7.20 | 7.18 | 0.70 | 6.60 | 1.20 |
| 17 | 6.39 | 10.24 | 4.70 | 9.70 | 1.90 | 11.49 | 1.80 | 9.40 |
| 18 | 10.77 | 4.14 | 6.30 | 3.00 | 11.21 | 7.66 | 5.00 | 7.00 |
| 19 | 2.05 | 1.65 | 4.00 | 0.70 | 2.14 | 3.13 | 0.10 | 3.70 |
| 20 | 2.74 | 3.48 | 1.40 | 4.30 | 2.08 | 1.90 | 2.20 | 5.80 |
| 21 | 0.69 | 4.45 | 3.60 | 12.80 | 1.41 | 5.30 | 0.60 | 13.30 |

CFP: Color Fundus Photography, mo: months, OCT: Optical Coherence Tomography, OD: Right eye, OS: Left eye
